# Impact of a naturally occurring hepatitis B virus genotype D-specific core-promoter mutation on viral replication

**DOI:** 10.1099/jgv.0.002225

**Published:** 2026-02-19

**Authors:** Masatake Kanai, Tadasu Shin-I, Tomoko Date, Aiko Sakai, Masashi Mizokami, Masaya Sugiyama

**Affiliations:** 1 Department of Viral Pathogenesis and Controls, National Institute of Global Health and Medicine, Japan Institute for Health and Security, Tokyo, Japan; 2 Laboratory of Macromolecular Biochemistry, Department of Chemistry for Life Sciences and Agriculture, Faculty of Life Science, Tokyo University of Agriculture, Tokyo, Japan; 3 Cellular & Molecular Biotechnology Research Institute, National Institute of Advanced Industrial Science and Technology, Ibaraki, Japan

**Keywords:** G1757A, A1762T/G1764A, G1764T/C1766G, genotype D, hepatitis B virus, viral replication

## Abstract

Hepatitis B virus (HBV) infects human populations worldwide. HBV strains are classified into 10 genotypes, of which the HBV genotype D (HBV/D) infection is particularly prevalent in several countries. The HBV core promoter regulates viral replication and transcription, and the naturally occurring A1762T/G1764A double mutation (CP1) in the core promoter accelerates HBV replication. Previous clinical studies showed that a new core-promoter mutation, G1764T/C1766G (CP2), is frequently observed in genomes containing the G1757A substitution, which is unique to HBV/D; however, CP2 is not observed in genomes containing the 1762T/1764A double mutation. In this study, we found that the CP2 mutation dramatically increased viral replication and transcription efficiency in two cell lines; the degree of stimulation was comparable to that induced by CP1. Introduction of the 1757A substitution reduced the increase in viral replication induced by the CP1 mutation. By contrast, the addition of the 1757A substitution significantly increased the effect of the CP2 mutation. The transcriptional activity of CP1 was decreased by the 1757A substitution, due to a reduction in HNF1 binding affinity, suggesting that 1757G is an important component of the HNF1 binding consensus sequence. The HBV/D-specific CP2 mutation creates a binding site for the transcription factor HNF3, thereby increasing its transcriptional activity. HBX proteins containing substitutions reflecting the two types of core-promoter mutations did not affect the efficiency of viral replication. Therefore, we hypothesize that the introduction of the CP2 mutation represents a survival strategy for HBV/D, allowing it to escape the effect of the 1757A substitution.

**Impact Statement**

Hepatitis B virus (HBV) infects ~250 million people worldwide. HBV genotype D (HBV/D), the most prevalent genotype worldwide, contains the unique substitution G1757A, often in combination with the G1764T/C1766G (CP2) double mutation. Another double mutation, A1762T/G1764A (CP1), is rare in HBV/D with G1757A but is common in other genotypes. These mutations are associated with clinical outcomes. In this study, the CP2 mutation increased viral replication efficiency as effectively as the CP1 mutation. Replication of the CP1 mutant was decreased, whereas that of the CP2 mutant was increased, by the addition of the 1757A substitution. These significant changes were based on differences in transcription factor binding: the 1757A substitution inhibited binding of the transcription factor, HNF1, to the CP1 mutant, but did not affect binding to the CP2 mutant. These mutation patterns in HBV/D may represent a survival strategy that allows the virus to spread more widely.

## Introduction

The major cause of hepatocellular carcinomas (HCC) worldwide is infection with the hepatitis B virus (HBV) and/or hepatitis C virus [1,2]. Chronic infection with HBV is the most important risk factor for HCC in humans: more than 254 million people carry the virus [2]. Given that only a fraction of people chronically infected with HBV will eventually develop HCC, it is likely that specific viral characteristics contribute to HBV-induced hepatocarcinogenesis. The contribution of HBV to the pathogenesis of liver cancer is multifactorial and complicated by the identification of mutant variants of HBV that modulate carcinogenesis [3].

HBV, a member of the hepadnavirus family, is a DNA virus with a partially double-stranded DNA genome that adopts a covalently closed circular conformation. HBV encodes four overlapping ORFs encoding surface proteins (HBsAg), core proteins (HBe/HBcAg), a polymerase and the X protein (HBx) [4]. Therefore, mutations in the HBV genome can alter the sequences or expression levels of multiple proteins. A double mutation in the HBV genome, an adenine-to-thymine transversion at nt 1762 and a guanine-to-adenine transition at nt 1764 (A1762T/G1764A), has been observed in tumours [5,6]. This region of the HBV genome contains a sequence that is a component of both the base core promoter (BCP) and the HBV X gene; the double mutation at nt 1762 and 1764 also changes codons 130 and 131 of the X gene. These mutations are associated with increased severity of HBV infection and cirrhosis [5,6]. In terms of virological characteristics, the 1764A single mutant replicates less efficiently than the wild-type, whereas the 1762T mutant induces faster production of progeny virus than the 1762T/1764A double mutant [7].

HBV is classified into ten genotypes, and those genotypes are further subdivided into subgenotypes on the basis of phylogenetic relationships [8,9]. Extensive evidence suggests that HBV genotypes/subgenotypes influence the pathogenesis of liver diseases in terms of acute, fulminant or chronic [10,14]. HBV genotype D (HBV/D) infects populations worldwide; the most prevalent subgenotype is D1 [15]. Two independent groups reported novel BCP mutations specific to HBV/D [16,17]. Triple mutations, a guanine-to-adenine transition at nt 1757, a guanine-to-thymine transversion at nt 1764 and a cytosine-to-guanine transversion at nt 1766 (G1757A/G1764T/C1766G), are unique to, and frequently observed in, the HBV/D genome; the 1762T/1764A double mutation has also been detected in HBV/D strains [16,17]. The 1757A substitution is common (55 % of HBV/D strains [17]) and, therefore, has originated in HBV/D. Patients infected with HBV/D carrying the 1757A substitution usually also harbour 1764T/1766G mutations, whereas the 1762T/1764A double mutations are found more frequently in combination with 1757G [16,17]. The relationship and detailed mechanism among 1757/1762/1764/1766 mutations remain unclear.

In this study, we examined the effects of HBV/D-specific mutations (1757A, 1764T and 1766G) on viral replication and HBV antigen production. The results indicate that the effect of the 1762T/1764A double mutation on viral replication can be inhibited by 1757A, whereas the 1757A mutation is beneficial for the replication of constructs containing 1764T/1766G. These differences are a consequence of the differential effects of these mutations on the binding affinities of hepatocyte nuclear factor (HNF) transcription factors.

## Methods

### Plasmid constructs

**Fig. 1.**
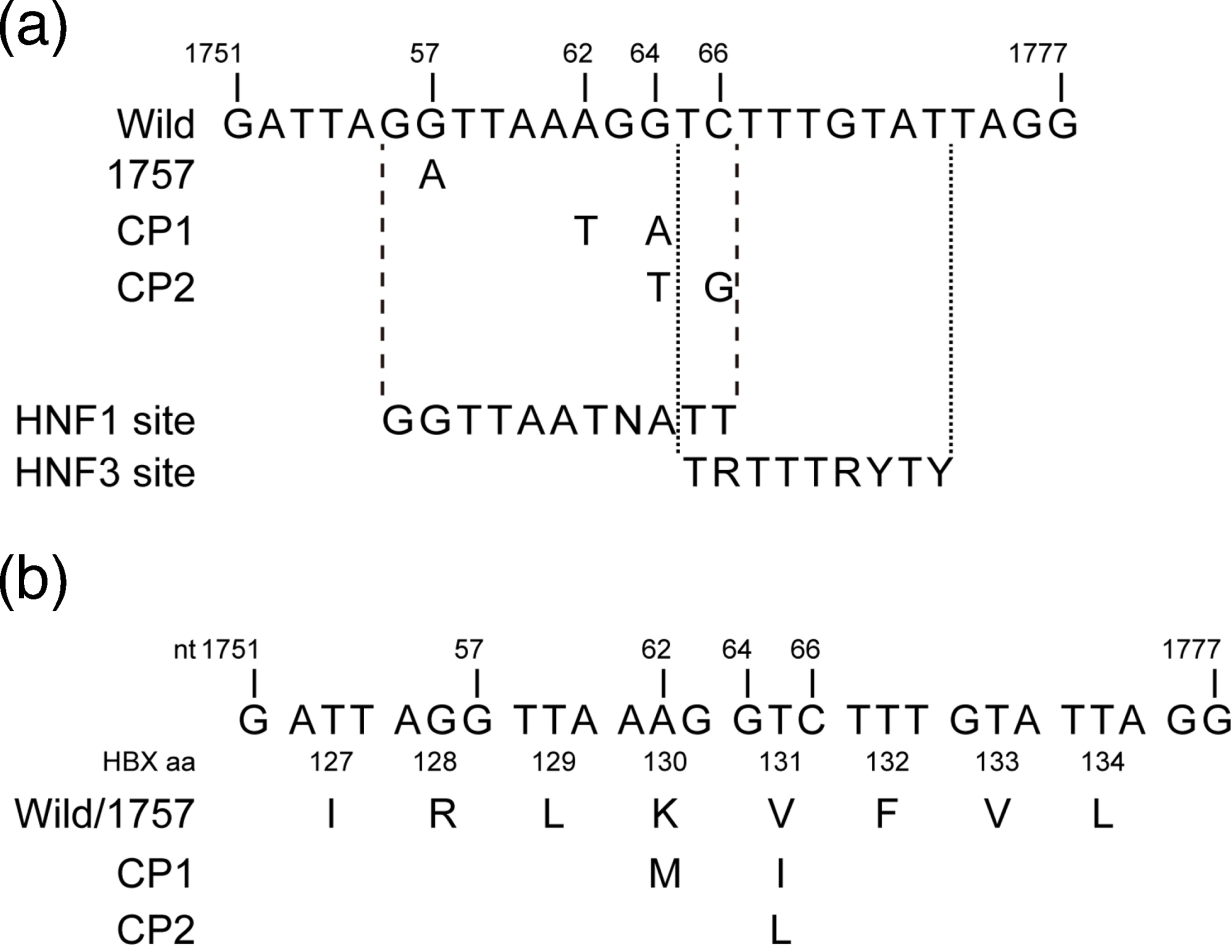
Schematic illustration of the HBV core promoter. (**a**) Comparison of regions within the core-promoter sequences harboured by the constructs used in this study. The consensus sequence of the transcription factor-binding site is described below [32,36,38]. V=A/G/C; W=A/T; R=A/G; K=G/T; Y=T/C. (**b**) The amino-acid sequence of the X protein is also shown. The locations of nucleotide and amino-acid mutations are displayed under the wild-type sequence. nt, nucleotide number of HBV genome; HBX_aa, amino-acid number of HBX.

Plasmid carrying HBV genomes of 1.24-fold length, pUC19/D_IND60 (AB246347, subgenotype D1), was described previously [18]. Fig. 1 shows the point mutation in the core promoter used in this study. The 1762T/1764A and 1764T/1766G double mutations were named CP1 and CP2, respectively. The target mutations were introduced into the plasmid by site-directed mutagenesis. The constructs used in the luciferase assays were generated by amplifying the core promoter using primers with linkers containing *Kpn*I or *Hind*III sites and then cloning the resulting amplicons into the pGL4 luciferase vector (Promega, Madison, WI, USA). In addition, we generated expression plasmids encoding the hepatitis B X protein (HBx) gene. The HBx gene was inserted into pcDNA3.1/Hyg (Invitrogen Corp., Carlsbad, CA, USA) and fused to the myc tag. These plasmids were capable of driving the synthesis of the wild-type, 1762T/1764A (K130M/V131I) or 1764T/1766G (V131L) HBx proteins from the cytomegalovirus (CMV) promoter. Cloned HBV DNA sequences were confirmed on an ABI 3100 automated sequencer (Applied Biosystems, Foster City, CA, USA) using Prism Big Dye v3.1 (Applied Biosystems).

### Cell culture and transfection

HuH7 and HepG2 cells were cultured in Dulbecco's Modified Eagle Medium (DMEM) supplemented with 10 % FBS. For the standard replication assay, dishes (10 cm in diameter) were seeded with 1×10^6^ cells. After 16 h of incubation, HuH7 or HepG2 cells were transfected with 5 µg of pUC19-based replication construct, with or without the CMV-driven expression construct, using the Fugene 6 transfection reagent (Roche Diagnostics, Indianapolis, IN, USA), and then harvested 72 h later. Transfection efficiency was measured by co-transfection with 0.5 µg of a reporter plasmid expressing secreted alkaline phosphatase (SEAP). SEAP activity was then measured in the culture supernatant and normalized with subsequent SEAP measurement from culture supernatant using a SEAP reporter assay kit (TOYOBO). The range of SEAP activity values was within 10 %. If the value of even one sample is over 10%, the experiment was omitted to match the background condition of the experiment. All data are based on the results of at least three independent experiments.

### Determination of HBV markers

Hepatitis B surface antigen (HBsAg) and hepatitis B e-antigen (HBeAg) levels were determined by chemiluminescence using commercial assay kits (Fujirebio Inc., Tokyo, Japan). The product of the HBV core gene (the core protein) was measured in an enzyme immunoassay using a monoclonal antibody (HB50) that specifically recognizes the SPRRR repeats in the arginine-rich domain of the core protein [19].

### Preparation of RNA

Transfected cells were lysed with ISOGEN (Nippon Gene, Tokyo, Japan). The lysate was supplemented with chloroform, incubated for 15 min on ice and centrifuged at 22,000 ***g*** for 15 min. The aqueous phase was removed and precipitated with isopropanol. RNA was pelleted by centrifugation, washed with ethanol and dissolved in 50 µl of water.

### Southern and Northern blot hybridizations

Southern and Northern blot hybridizations were performed with a full-length probe specific for each genotype/subgenotype, as described previously [20]. In brief, for the collection of core-associated HBV-DNA, cells are suspended in 1.5 ml of lysis buffer containing 50 mM Tris-HCl (pH 7.4), 1 mM EDTA and 1 % NP-40. Nuclei were pelleted by centrifugation at 4 °C at 22,000 ***g*** for 5 min. The supernatant was adjusted to 6 mM MgOAc2 and treated with 200 µg ml^−1^ DNase I and 100 µg ml^−1^ RNase A for 2 h at 37 °C. The reaction was stopped by the addition of EDTA to a final concentration of 10 mM, and then, the mixture was incubated for 10 min at 65 °C. Proteins in the sample were digested with 200 µg ml^−1^ Proteinase K, 1 % SDS and 100 mM NaCl for 2 h at 37 °C. Nucleic acids were purified by extraction with phenol–chloroform (1:1) and precipitation with ethanol after the addition of NaOAc and glycogen. Isolated core-associated HBV-DNA was separated by electrophoresis on a 1.2 % agarose gel and total RNA (20 µg) was separated on a 1 % agarose-formaldehyde gel. DNA and RNA were transferred onto a positively charged nylon membrane (Roche Diagnostics, Germany) and hybridized with either the alkaline phosphatase-labelled full-length HBV fragment or a 1.3 kb glyceraldehyde-3-phosphate dehydrogenase (GAPDH). cDNA fragments were generated with a Gene Images AlkPhos direct labelling system (Amersham Biosciences, UK), according to the manufacturer’s instructions. The hybridization was performed at 55 °C and detected with CDP-star (Amersham Biosciences, UK) according to the manufacturer’s instructions. Signals were analysed by the LAS-1000 image analyser (Fuji Photo Film, Japan)

### Detection of extracellular HBV

Extracellular HBV was quantified by real-time detection PCR as previously reported [18]. Briefly, 10 µl of the eluted sample was amplified in a total reaction volume of 50 µl containing 2×TaqMan Universal Master Mix II (Applied Biosystems, Foster City, CA), a forward primer (HBV-S190F; GCT CGT GTT ACA GGC GGG), a reverse primer (HBV-S703R; GAA CCA CTG AAC AAA TGG CAC TAG TA) and a TaqMan probe (HBSP2G; FAM-ATG TTG CCC GTT TGT CCT CTA ATT CCA G-TAMRA).

PCR amplification and signal detection were carried out using the LightCycler 480 system II (Roche Diagnostics GmbH, Mannheim, Germany). The thermal cycling protocol consisted of an initial uracil-N-glycosylase activation step at 50 °C for 2 min, followed by enzyme activation at 95 °C for 10 min. Subsequently, 35 cycles of amplification were performed, each comprising denaturation at 95 °C for 20 s and annealing/extension at 60 °C for 1 min. Quantification was achieved using a standard curve generated from serial dilutions of a cloned HBV plasmid with a known copy number.

### Luciferase assay

To confirm the ability of firefly luciferase activity to reliably measure core promoter transcription, established cell lines were plated in 96-well plates (10^4^ cells per well) and then transfected with 50 ng of pGL4.74-Renilla luciferase and 50 ng of either a pGL4-D1CP-Firefly luciferase construct containing a 375 bp core-promoter sequence (nt 1413–1788) upstream of the precore gene start codon or the pGL4 control plasmid. NC, which contained no HBV sequence of the pGL4 control plasmid, was used as a negative control. PC, which contained the HNF1 and HNF3 consensus sequence, was used as a positive control (Fig. 1 and Table 1). In addition, 20 ng of pcDNA3 or pcDNA3/HNF3A, kindly provided by Dr. Robert H. Costa (University of Illinois), was co-transfected into cells together with Renilla and firefly luciferase plasmids to assess the consistent effect of HNF3 on each HBV core promoter sequence. Cells were harvested and lysed in 75 µl of luciferase assay reagent 48 h after transfection. Luciferase activity was quantified using the Dual-Glo luciferase reporter assay system (Promega, Madison, WI, USA) on an Lmax microplate luminometer (Molecular Devices, Sunnyvale, CA, USA).

**Table 1.** Probes and biotin-labelled primer

Probe	5′-linker	HBV core promoter (nt/1752–1779)	3′-linker
Wild	nnnnnnnnnnnnnnnnggggag	ATTAGGTTAAAGGTCTTTGTATTAGGAG	nnnnnnnnnn gaaaggatgggtgcgacgcg
1757G/1762T	nnnnnnnnnnnnnnnnggggag	ATTAGGTTAA**T**GGTCTTTGTATTAGGAG	nnnnnnnnnn gaaaggatgggtgcgacgcg
1757G/1764A	nnnnnnnnnnnnnnnnggggag	ATTAGGTTAAAG**A**TCTTTGTATTAGGAG	nnnnnnnnnn gaaaggatgggtgcgacgcg
1757 G/CP1	nnnnnnnnnnnnnnnnggggag	ATTAGGTTAA**T**G**A**TCTTTGTATTAGGAG	nnnnnnnnnn gaaaggatgggtgcgacgcg
1757A	nnnnnnnnnnnnnnnnggggag	ATTAG**A**TTAAAGGTCTTTGTATTAGGAG	nnnnnnnnnn gaaaggatgggtgcgacgcg
1757A/1762T	nnnnnnnnnnnnnnnnggggag	ATTAG**A**TTAA**T**GGTCTTTGTATTAGGAG	nnnnnnnnnn gaaaggatgggtgcgacgcg
1757A/1764A	nnnnnnnnnnnnnnnnggggag	ATTAG**A**TTAAAG**A**TCTTTGTATTAGGAG	nnnnnnnnnn gaaaggatgggtgcgacgcg
1757 A/CP1	nnnnnnnnnnnnnnnnggggag	ATTAG**A**TTAA**T**G**A**TCTTTGTATTAGGAG	nnnnnnnnnn gaaaggatgggtgcgacgcg
1757G/1764T	nnnnnnnnnnnnnnnnggggag	ATTAGGTTAAAG**T**TCTTTGTATTAGGAG	nnnnnnnnnn gaaaggatgggtgcgacgcg
1757G/1766G	nnnnnnnnnnnnnnnnggggag	ATTAGGTTAAAGGT**G**TTTGTATTAGGAG	nnnnnnnnnn gaaaggatgggtgcgacgcg
1757 G/CP2	nnnnnnnnnnnnnnnnggggag	ATTAGGTTAAAG**T**T**G**TTTGTATTAGGAG	nnnnnnnnnn gaaaggatgggtgcgacgcg
1757A/1764T	nnnnnnnnnnnnnnnnggggag	ATTAG**A**TTAAAG**T**TCTTTGTATTAGGAG	nnnnnnnnnn gaaaggatgggtgcgacgcg
1757A/1766G	nnnnnnnnnnnnnnnnggggag	ATTAG**A**TTAAAGGT**G**TTTGTATTAGGAG	nnnnnnnnnn gaaaggatgggtgcgacgcg
1757 A/CP2	nnnnnnnnnnnnnnnnggggag	ATTAG**A**TTAAAG**T**T**G**TTTGTATTAGGAG	nnnnnnnnnn gaaaggatgggtgcgacgcg
Negative control	nnnnnnnnnnnnnnnnggggag	NNNNNNNNNNNNNNNNNNNNNNNNNNN	nnnnnnnnnn gaaaggatgggtgcgacgcg
Positive control -	nnnnnnnnnnnnnnnnggggag	TRTTTRYTY	nnnnnnnnnn gaaaggatgggtgcgacgcg
Positive control (HNF1)	nnnnnnnnnnnnnnnnggggag	GGTTAATGATT	nnnnnnnnnn gaaaggatgggtgcgacgcg
Universal Primer	Biotin-cgcgtcgcacccatcctttc		

Probe name shows each mutation position.

The underlined nucleotides are mutations relating to the present study.

The universal primer, which complements the 3′-linker of the probe, is applied to each probe to generate double-stranded DNA probes during the PCR reaction.

The positive control for HNF3 or HNF1 contains their consensus sequences (Fig. 1).

*N*=random; R=A/G; Y=T/C.

### Quantification PCR analysis of HBV RNA

Precore mRNA (pcRNA) and pre-genomic RNA (pgRNA) levels were quantified by real-time quantitative PCR. Total cellular RNA and DNA were isolated from transfected cells using TRIzol reagent (Invitrogen, Carlsbad, CA, USA) according to the manufacturer’s instructions. Extracted RNA was treated with RQ1 RNase-free DNase (Promega, Madison, WI, USA) at 37 °C for 1 h to eliminate residual DNA. RNA concentration and purity were assessed by spectrophotometric analysis. For cDNA synthesis, 1 µg of DNase-treated RNA was reverse-transcribed using random hexamer primers (Biodynamics, Buenos Aires, Argentina) and M-MLV reverse transcriptase (Promega, Madison, WI, USA). pcRNA and pgRNA were then quantified as previously described [21,22]. Briefly, the resulting cDNA was subjected to two independent PCR reactions using a common antisense primer (5′-GGAAAGAAGTCAGAAGGCAA-3′; nt 1974–1955) in combination with either a pcRNA-specific sense primer (5′-GGTCTGCGCACCAGCACC-3′; nt 1796–1813) or a sense primer that detects both pcRNA and pgRNA transcripts (5′-CACCTCTGCCTAATCATC-3′; nt 1826–1843). The level of pgRNA was calculated by subtracting pcRNA levels from the total core promoter-directed transcripts comprising pcRNA and pgRNA. This calculation is based on the assumption that the signal obtained with the latter primer set represents the combined abundance of pcRNA and pgRNA transcribed from the core promoter. GAPDH mRNA was amplified in parallel and used as an internal control for normalization. The primer sequences for GAPDH amplification were as follows: sense, 5′-GAAGGTGAAGGTCGGAGTC-3′; antisense, 5′-GAAGATGGTGATGGGATTTC-3′. For quantitative analysis, serial dilutions of an HBV replication-competent plasmid were used to generate standard curves. To exclude amplification derived from contaminating DNA, PCR reactions were routinely performed in the absence of reverse transcriptase as a negative control. Cell number was estimated by quantifying *β*-globin gene levels in extracted genomic DNA using the LightCycler Control Kit DNA (Roche Diagnostics GmbH, Mannheim, Germany), and the results were normalized to 1×10^4^ cells.

### Transcription factor binding assay

The activities of HNF1 and HNF3 were then assessed using the TransAM flexi Transcription Factor Assay Kit (Active Motif, Rixensart, Belgium) in accordance with the manufacturer’s instructions. In summary, 5 µg of nuclear extract was applied to individual wells of a 96-well plate that had been pre-coated with oligonucleotides obtained from HBV/D sequences (Table 1). Following a 1-h incubation with gentle agitation at room temperature, the wells were washed thrice with the provided washing buffer and subsequently incubated with anti-HNF1 or anti-HNF3 antibodies (1:1,000 dilution) for 1 h at 20 °C. Following a further three washes, the wells were then incubated with horseradish peroxidase-conjugated secondary antibody (1:1,000 dilution) for a period of 1 h. This was followed by the addition of 100 µl of developing solution. Following a 5-min incubation period, the reaction was terminated by the addition of 100 µl of stop solution. Absorbance was measured at a wavelength of 450 nm, with a reference wavelength of 655 nm, using a microplate spectrophotometer. To further characterize the binding specificity of HNF1 and HNF3, competition assays were performed using free oligonucleotide probes. Nuclear extracts were preincubated with a 100-fold molar excess of free competitor probe prior to being applied to the wells coated with the detection probes. For the A1762T/G1764A (CP1) series, a free competitor probe containing the HNF1 consensus binding sequence was used, whereas for the G1764T/C1766G (CP2) series, a free competitor probe containing the HNF3 consensus binding sequence was employed. The nuclear extract–competitor probe mixtures were then added to the respective probe-coated wells and processed as described above.

### Immunoblot analysis

Immunoblot analysis was performed as previously described [23]. Briefly, grown cells were scraped on day 2 post-transfection, and the cell pellet was lysed with CellLytic^™^-M buffer (Sigma-Aldrich, St. Louis, MO, USA). Proteins from 10 µl of lysate were separated on 0.1 % SDS/15 % gradient polyacrylamide gels and then transferred to a Hybond ECL nitrocellulose membrane (GE Healthcare). An HRP-conjugated mouse monoclonal anti-myc antibody (Invitrogen, Carlsbad, CA) was applied to the membrane, visualized with the ECL Plus detection kit (GE Healthcare), and detected on an LAS3000 imaging system.

### Statistical analysis

Data are presented as the mean±sd of at least three independent experiments. All statistical analyses were performed using SPSS 20 and GraphPad Prism 10 software. The results were analysed using Student’s unpaired two-tailed t-test or one-way ANOVA. A value of *P*<0.05 was regarded as statistically significant (*P<0.05).

## Results

### Effect of the CP1 or CP2 mutation on viral replication in the presence or absence of the 1757A substitution

**Fig. 2.**
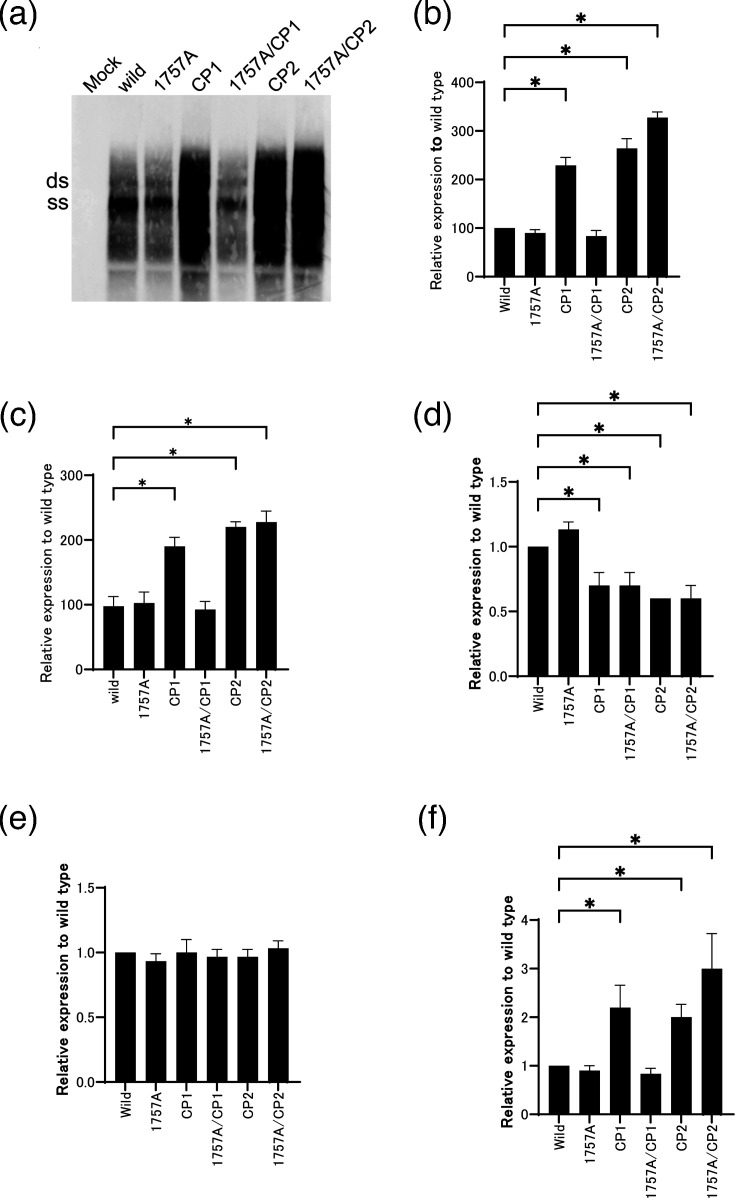
Influence of core-promoter mutations on viral replication and antigen production. Replication constructs harbouring the core-promoter mutations were transfected into HuH7 cells to compare the levels of viral replication and antigen production with those of the wild-type clone. (**a**) Viral replication levels were examined by Southern blotting with core-associated HBV DNA in HuH7 cells. Replication efficiency was compared by quantifying the intensity of the band using the Multi Gauge software (Fuji Film, Tokyo, Japan). (**b**) The intensity of the SS band on the Southern blot was quantified and plotted relative to the wild-type control. (**c**) Extracellular HBV DNA was quantified by real-time PCR. Core-associated HBV DNA was extracted from the cell culture supernatant of each dish. The expression levels of (**d**) HBeAg and (**e**) HBsAg or (**f**) intracellular HBcAg are shown relative to the wild-type. The experiments were independently repeated three times, and representative Southern blots are shown. Bars represent the averages±sd of the data obtained in three independent experiments. The transfection efficiency in all experiments was monitored by co-transfection of a SEAP expression vector. *P<0.05 (relative to wild-type).

To investigate how HBV/D-specific mutations affect the activity of the core promoter, we transfected wild-type or mutant HBV constructs into HuH7 cells. The density of HBV DNA band was compared between constructs by Southern blotting (Fig. 2a, b). The replication rate of the CP1 mutant was approximately twofold higher (as reported previously); however, this increase was diminished by the introduction of the 1757A substitution (1757 A/CP1 lane). Viral replication of the CP2 mutant was ~2.5-fold higher than that of the wild-type clone, and the introduction of 1757A to CP2 further increased replication. Similar results were observed for the extracellular HBV DNA levels shown in Fig. 2c. The level of HBeAg was modestly reduced in all mutants, except for the mutant harbouring 1757A alone (Fig. 2d), whereas the level of HBsAg expression was similar for all constructs (Fig. 2e). Levels of intracellular hepatitis B core antigen (HBcAg) correlated with levels of viral replication (Fig. 2f). We also confirmed these observations in the HepG2 cell line, suggesting that the effects of the CP1 and CP2 mutations are independent of the host cell line (data not shown).

### The role of individual mutations in the CP region on viral replication

**Fig. 3.**
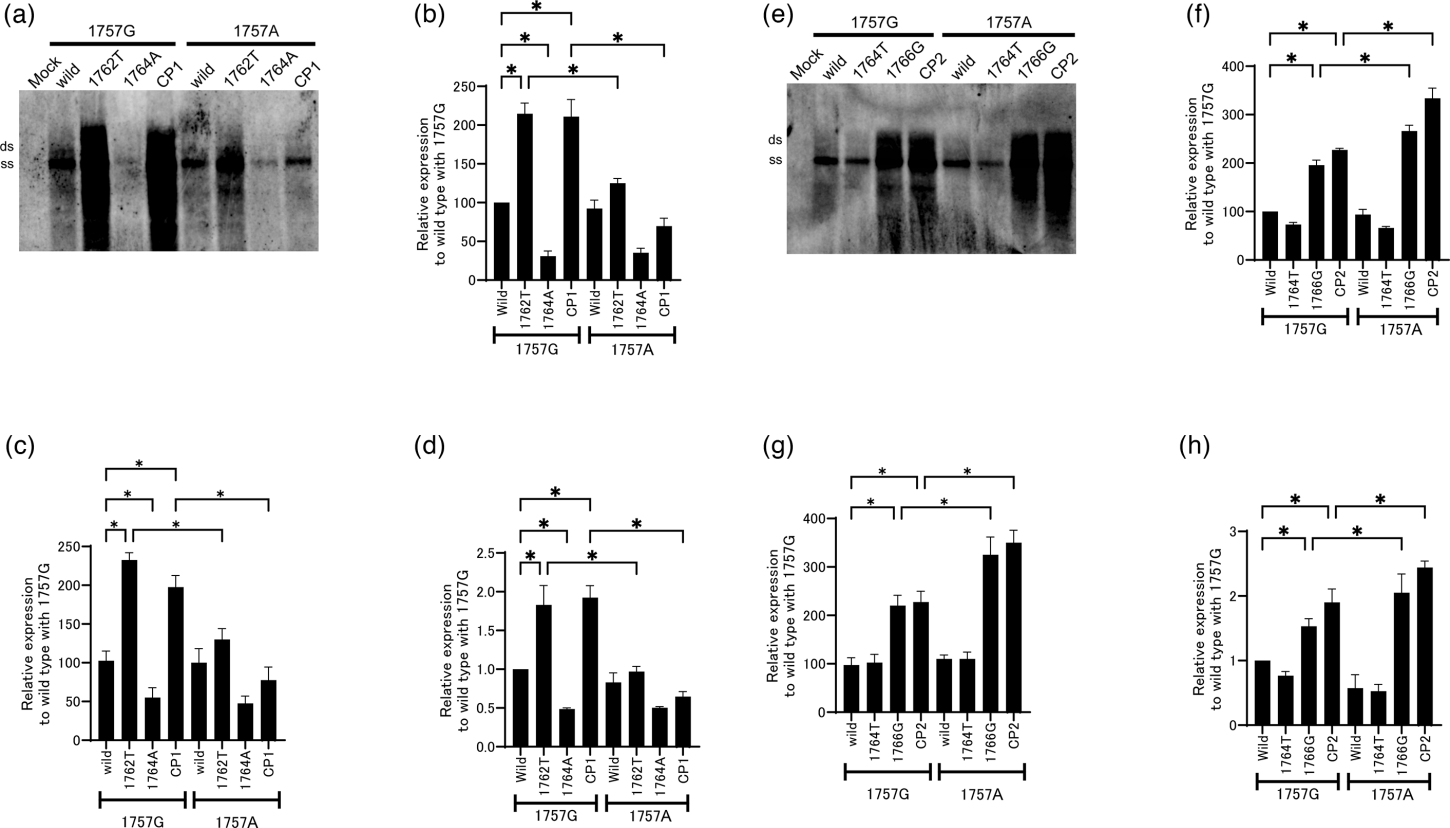
The effect of each mutation, both individually and in combination, on the viral replication of CP1 and CP2. To determine the effect of the individual mutations in HBV/D (1757A, 1762T, 1764A, 1764T and/or 1766G), these mutations were introduced into wild-type clones. (**a, e**) Each clone was transfected into HuH7 cells, and cell lysates were collected 3 days later. Isolated core-associated HBV DNA was analysed by Southern blotting. The replication efficiency was compared by quantifying the intensity of the band using the Multi Gauge software. (**b, f**) The intensity of the SS band on the Southern blot was quantified and plotted relative to the wild-type control. (**c, g**) Extracellular HBV DNA was quantified by real-time PCR. Core-associated HBV DNA was extracted from the cell culture supernatant of each dish. (**d, h**) Quantitation of intracellular HBcAg expressed by each clone, determined by EIA. Bars represent the averages±SD of the data obtained in three independent experiments. The transfection efficiency in all experiments was monitored by co-transfection of a SEAP expression vector. *P<0.05 (comparisons were made between the wild-type and other 1757G strains. The same mutant clones were also compared with and without 1757A).

We separated the CP1 double mutation (1762T/1764A) into individual mutations, which we then tested in the presence or absence of the 1757A substitution to analyse their effects on viral replication. As reported previously, the 1762T mutation alone conferred the most effective viral replication [7], whereas the replication efficiency of 1764A alone was reduced to less than wild-type level (*P*<0.05) (Fig. 3a, b). When introduced into the constructs, the 1757A substitution reduced the viral replication efficiency of 1762T and CP1 mutants. A similar trend was observed in the extracellular HBV DNA measurements (Fig. 3c). The intracellular amount of HBcAg exhibited a trend similar to that observed in the Southern blot analysis (Fig. 3d).

Next, the replication efficiency of the CP2 mutants was analysed by investigating the effect of the individual mutations, with or without 1757A. The 1764T mutant, in the absence or presence of the 1757A mutation, exerted no influence on viral replication (Fig. 3e, f). Conversely, the 1766G mutant exhibited a heightened replication efficiency. The impact of the 1766G mutation was augmented by the introduction of the 1757A mutation. A comparable effect was observed in the CP2 mutant. Comparable extracellular HBV DNA levels were detected, as shown in Fig. 3g. The expression levels of intracellular HBcAg were correlated with the levels of viral replication, as determined by Southern blotting (Fig. 3h).

### The transcription activity of the CP1 and CP2 mutation with or without the 1757A mutation

**Fig. 4.**
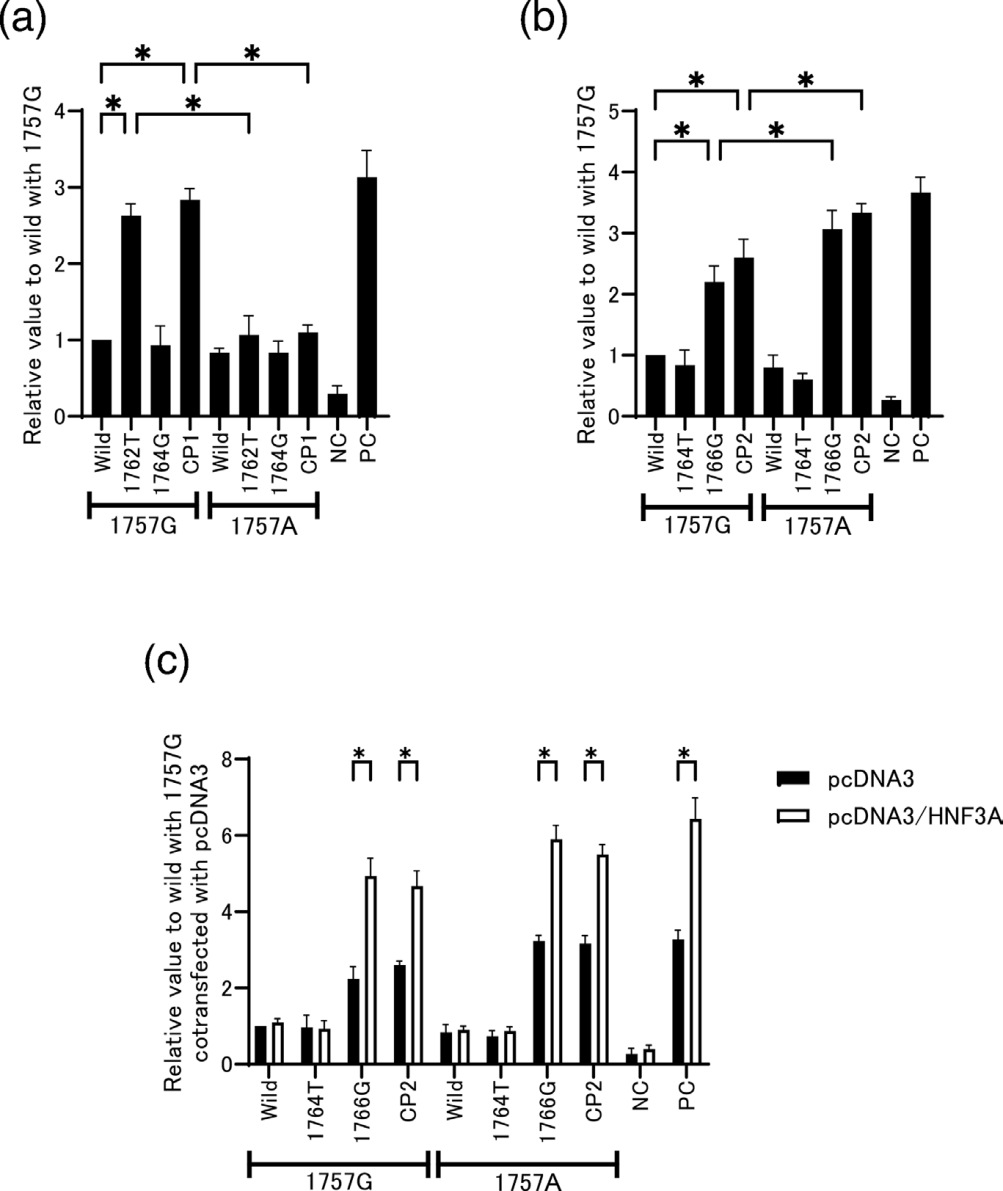
Effect of HNFs on the transcriptional activity of the core-promoter mutants. Reporter plasmids containing core-promoter inserts with specific mutations and a control plasmid were transfected into HuH7 cells. (**a**) CP1 series and (**b**) CP2 series, each with or without the G1757A mutation. (**c**) In the CP2 series, the HNF3 expression plasmid (pcDNA3/HNF3A) was co-transfected under the same conditions as in (**b**). Luciferase activity was examined 48 h after transfection. Transfection efficiency was normalized against Renilla luciferase activity. The positive control (PC) used in (**a**) contains the HNF1 consensus binding sequence, whereas the PC used in (**b, c**) contains the HNF3 consensus binding sequence. The negative control (NC) is an empty vector. *P<0.05 (comparisons were made between the wild-type and other 1757G strains. The same mutant clones were also compared with and without 1757A).

Next, we investigated the effect of the CP1 and CP2 mutation on transcriptional activity with or without the 1757A mutation. To investigate the effects of these mutations on the transcriptional activity of HuH7 cells under their endogenous transcription factor, we cloned the core promoter regions of the constructs into the pGL4 luciferase expression vector. Along with Fig. 3 data, the transcriptional activity of the 1762T and CP1 mutant was diminished by the addition of the 1757A mutation (Fig. 4a). Conversely, in the context of the CP2 mutation, the presence of the 1757A mutation further enhanced the transcriptional activation induced by the 1766G and CP2 mutations, compared to the activation observed in the absence of 1757A (Fig. 4b). To confirm the effect of HNF3 on CP2 mutations, an HNF3 expression plasmid was co-transfected under the same conditions as those used in (Fig. 4b, c). Consistent with the results shown in Fig. 4b, the transcriptional activities of the 1766G and CP2 mutations were further upregulated in the presence of both the 1757G and 1757A mutations. These findings suggest that the 1757A and CP2 mutations may influence viral replication efficiency by modulating transcriptional activity.

### Regulation of pre-genomic RNA levels by the CP mutation

**Fig. 5.**
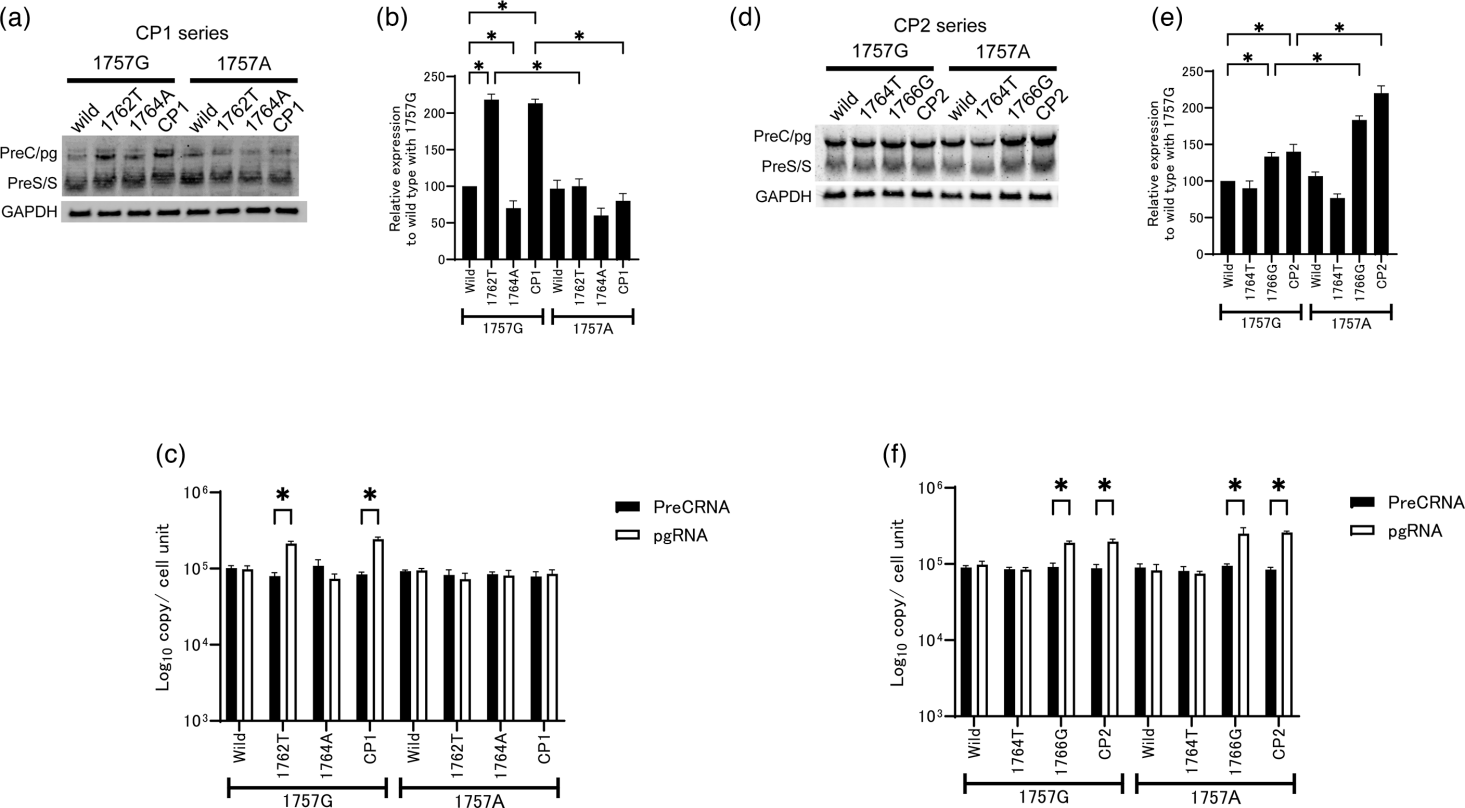
Comparison of HBV RNA expression levels between the CP1 and CP2 mutants. Total RNA was collected from HuH7 cells transfected with the indicated constructs. Northern blot analysis was performed after separation of RNA on denaturing gels. (**a**) Constructs containing the CP1 mutation in combination with other mutations were transfected into cells. (**d**) Constructs containing the CP2 mutation in combination with other mutations were transfected into cells. The transcription efficiency was compared by quantifying the intensity of the PreC/pg mRNA band using the Multi Gauge software. (**b, e**) The intensity of the SS band on the Southern blot was quantified and plotted relative to the wild-type control. (**c, f**) PreC RNA and pgRNA were quantified by real-time PCR using total RNA extracts. pgRNA levels were calculated by subtracting PreC RNA levels, determined by a PreC-specific PCR, from the total core promoter-directed transcripts quantified by the PreC/pgRNA PCR assay. To account for differences in cell number, RNA levels were normalized on a per-cell basis using genomic DNA quantification. The experiments were independently repeated three times, and representative Northern blots are shown. The transfection in all experiments was monitored by co-transfection of a SEAP expression vector. PreC/pg, precore and pre-genomic RNA; PreS/S, preS1-2 and S RNA; GAPDH, glyceraldehyde-3-phosphate dehydrogenase RNA. *P<0.05 (comparisons were made between the wild-type and other 1757G strains. The same mutant clones were also compared with and without 1757A).

To confirm this result of the transcription activity data, we used replication constructs to determine the levels of HBV-derived mRNA by Northern blotting and quantified real-time PCR. Changes at the transcriptional level were first assessed by measuring precore/pre-genomic (preC/pg) RNA levels. The expression of preC/pg RNA was increased in the 1762T and CP1 clones; however, this increase was attenuated by the additional introduction of the G1757A substitution (Fig. 5a, b). To further delineate the individual contributions of preC RNA and pgRNA, we performed PCR assays that quantified each transcript (Fig. 5c). Consistent with previous reports, pgRNA levels were markedly increased in the 1762T and CP1 clones. However, this enhancement was abolished by the additional introduction of the G1757A substitution. We next examined the effects of the 1764/1766 mutations in a similar manner. The 1766G and CP2 clone produced higher levels of preC/pg RNA than the wild-type clone, and the CP2 clone harbouring the G1757A substitution generated the highest levels of preC/pg RNA among all constructs tested (Fig. 5d, e). Separate quantification of preC RNA and pgRNA revealed a pronounced increase in pgRNA levels in the 1766G and CP2 clones, which was further enhanced by the introduction of the G1757A substitution (Fig. 5f). These transcriptional changes were well correlated with the results obtained from the viral replication assays and reporter assays.

### The binding affinity of HNF1 or HNF3 for the CP1 and CP2 mutant sequences, with or without the 1757A substitution

**Fig. 6.**
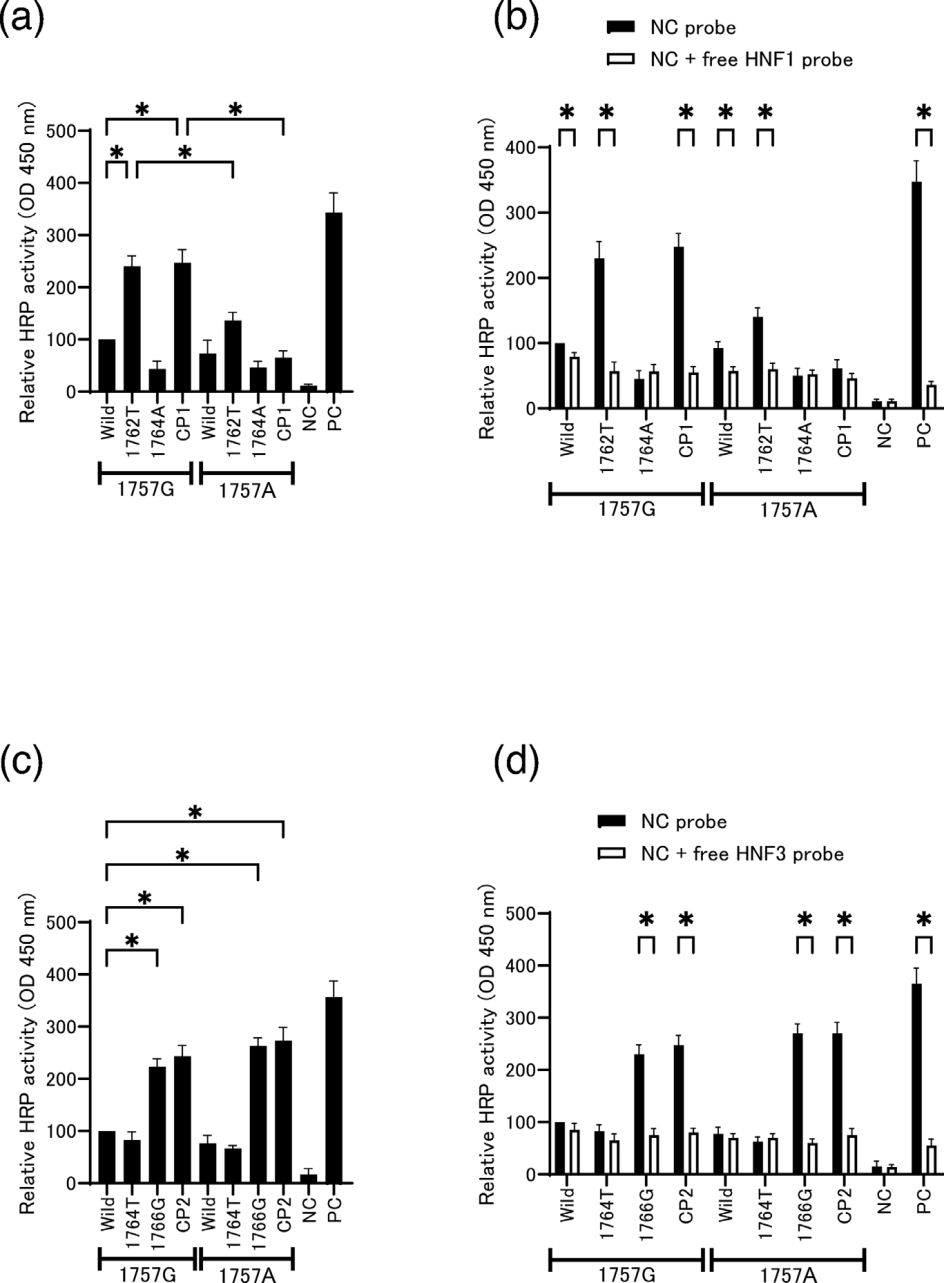
Transcription factor binding to the core promoter region. A transcription factor binding assay was performed to observe the binding of HNF proteins. Sequences of DNA probes are provided in Table 1. Biotin-tagged DNA probes containing the core promoter were pre-coated in a microplate. Nuclear extracts from HuH7 cells were applied to individual wells. (**a**) The core promoter probes containing mutations of the CP1 series and anti-HNF1 antibody were used for this assay. (**c**) The core promoter probes containing mutations of the CP2 series and anti-HNF3 antibody were used for this assay. Competition assays were performed using free oligonucleotide probes. Nuclear extracts were preincubated with a 100-fold molar excess of free competitor probe containing either (**b**) the HNF1 (CP1 series) or (**d**) HNF3 (CP2 series) consensus binding sequence before being applied to the probe-coated wells. Three independent experiments were performed. *P<0.05 (comparisons were made between the wild-type and other 1757G strains. The same mutant clones were also compared with and without 1757A).

To assess whether HNF1 or HNF3 binds to the region containing the HBV/D-specific mutation, we conducted transcription factor binding assays using wild-type and mutant DNA probes (Table 1) incubated with nuclear extracts from HuH7 cells. HNF1 binding was enhanced with the 1762T and CP1 mutant probes lacking the 1757A mutation, whereas the introduction of the 1757A mutation led to a marked reduction in HNF1 binding activity (Fig. 6a). In the CP2 series, both the 1766G and CP2 mutant probes, regardless of the presence of the 1757A mutation, exhibited higher HNF3 binding activity compared with the other probes (Fig. 6c). To verify these binding interactions, competition assays were performed using free oligonucleotide probes containing the consensus binding sequence for either HNF1 or HNF3. When nuclear extracts were preincubated with the free competitor probes, the binding signals observed in Fig. 6a, c were abolished, as shown in Fig. 6b, d. These results suggest that HNF1 binding to the CP1 mutant is negatively affected by the 1757A substitution, whereas in the case of the CP2 mutant, the 1757A mutation appears to exert a synergistic effect, further enhancing transcription factor binding.

### The effect of core-promoter mutations on the HBx protein

**Fig. 7.**
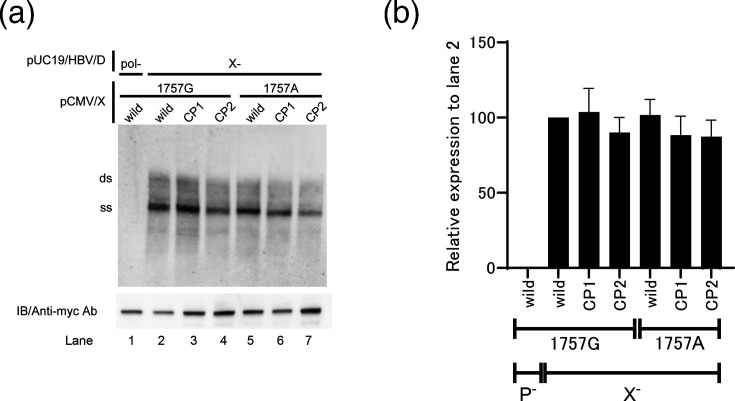
Effect of HBx mutations corresponding to the CP1 and CP2 mutations on viral replication. HBx-deficient wild-type pUC19/HBV/D constructs were co-transfected with myc-tagged pCMV/X constructs to compare viral replication levels in HepG2 cells. HBV polymerase-deficient constructs were used as a negative control. HBx mutants were constructed according to the mutation profile described below. (**a**) HBX expression was determined by immunoblotting (IB) using an anti-myc antibody. Viral replication levels were examined by Southern blotting with core-associated HBV DNA. The replication efficiency was compared by quantifying the intensity of the band using the Multi Gauge software. (**b**) The values shown under each lane are expressed relative to the wild-type with 1757G and X−. The experiments were independently repeated three times, and representative Southern blots are shown. The transfection efficiency in all experiments was monitored by co-transfection of a SEAP expression vector.

Because the HBx gene overlaps with the core promoter region, mutations within HBx have the potential to influence viral replication (Fig. 1b). To investigate this possibility, we generated an HBV genotype D replication construct containing a nonsense mutation in the HBx gene (pUC19/HBV/D/X−), together with a negative control construct carrying a loss-of-function mutation in the polymerase gene (pUC19/HBV/D/pol−). To assess potential trans-complementation effects on viral replication, these constructs were co-transfected into HepG2 cells along with pCMV/X expression plasmids encoding either wild-type or mutant HBx corresponding to the core promoter variants (wild-type, CP1 or CP2) (Fig. 1b). As a preliminary step, we evaluated the responsiveness of HepG2 cells to HBX expression (Fig. S1, available in the online Supplementary Material). Upon confirmation of both HBX sensitivity and dose-dependent effects, we proceeded with the subsequent experiments. Ectopic expression of HBx under the control of the CMV promoter did not enhance viral replication in any condition (Fig. 7a, b), indicating that the effects of the CP1 and CP2 mutations on replication are likely attributable to alterations within the core promoter sequence itself, rather than to changes in the overlapping HBx coding region.

## Discussion

Previous clinical studies show that core-promoter mutations are correlated with viral load, liver disease and responses to antiviral therapy [24,27]. Most of these papers focused on the 1762T/1764A double mutation and demonstrated an association between these mutations and the severity of liver disease [28,29]. The mechanism underlying the transcriptional effects of the CP1 mutation has been elucidated by several studies [7,30,32]. Both Elkady *et al*. and Sendi *et al.* identified the 1757A and 1764T/1766G mutations as HBV/D-specific substitutions and investigated their clinical significance in the context of chronic hepatitis B [16,17]. Therefore, our study focused on identifying the effects of the 1757A and CP2 mutations on viral replication and transcription.

Significant changes in viral replication were observed when the 1757A substitution was combined with CP1 or CP2, although 1757A alone did not alter viral replication efficiency. The effect of the 1757A substitution changed depending on the mutation pattern within the core promoter. When present in combination with CP1, the 1757A substitution suppressed viral replication, whereas it stimulated replication in the presence of the CP2 mutation. These differences in the effects of the 1757A substitution were a consequence of changes in transcriptional activity, which, in turn, resulted from differences in transcription factor binding. The sequence of the wild-type core-promoter sequence binds the transcriptional factor of HNF4, which is active in viral transcription [30]. The CP1 mutant has a markedly reduced affinity for HNF4, but this mutation creates a binding site for HNF1 [30,32]. The principal consensus sequence required for HNF1 binding comprises ~10 nt (1757–1766) within the core promoter region [30]. This site is more variable in HBV/D than it is in other HBV genotypes [16,17]. Consensus sequences of HNF1 binding sites contain 1757G. Transcription binding assay revealed that the HNF1 protein bound to the 1757G sequence in the 1762T mutant, whereas its binding affinity was diminished by the 1757A substitution. Furthermore, the transcriptional activity of pre-genomic RNA was also suppressed in a CP1 mutant harbouring the 1757A substitution. The 1757G may be a critical factor in determining HNF1 binding to the core promoter. These data suggest that the 1757A mutation negatively affects the increase in viral replication caused by the CP1 mutation, particularly the 1762T mutation. Therefore, HBV/D with a high frequency of the 1757A mutation may be less likely to exhibit the CP1 mutation [16,17].

Our findings indicate that the CP2 mutation confers a modest enhancement in viral replication efficiency compared to the wild-type construct, and this effect is further augmented by the 1757A substitution. While the 1766G mutation alone exerts a substantial impact on both viral replication and transcriptional activity, the combined CP2 mutations result in a markedly elevated replication level. In line with previous studies [16,17], the CP2 mutation appears to generate a *de novo* HNF3 binding site within the core promoter, and our *in vitro* assays confirmed the specific binding of HNF3 to this mutated region. Interestingly, the 1757A substitution, which is located distal to the HNF3 binding site, also contributed to increased replication. This suggests that its effect may not be mediated directly through HNF3, but rather through the involvement of another transcription factor, potentially forming a cooperative complex with HNF3. Further studies will be required to identify the factor(s) involved and to elucidate the mechanistic basis of this synergistic regulation.

The replication efficiency of the various mutants correlated with their levels of intracellular HBcAg expression. Our previous study also showed that HBcAg increases viral replication in a dose-dependent manner [23], suggesting that the amount of HBcAg might be a limiting factor in determining viral replication efficiency. In contrast, HBsAg levels remained unchanged across all mutants examined, likely due to the fact that the point mutations introduced into the core promoter region did not interfere with HBsAg production [33]. In agreement with the study by Zheng *et al*. [30], HBeAg expression was slightly reduced in the presence of CP1 and CP2 mutations. This reduction can be attributed to the shift in transcription factor binding – from HNF4 to HNF1/3 – which is known to decrease precore mRNA levels, the transcript responsible for HBeAg translation. These findings suggest that, similar to CP1, the CP2 mutation may contribute to reduced precore mRNA expression while enhancing pre-genomic RNA production, ultimately favouring viral replication.

The HBV genome contains multiple overlapping functional elements, including promoters, enhancers, packaging signals, direct repeats and protein-coding regions. As a result, the core promoter mutations analysed in this study also introduced amino acid changes in the overlapping HBx gene. The HBx protein is known to enhance viral replication [34], raising the possibility that these mutations may exert their effects through altered HBx function. Then, we ectopically expressed HBx variants harbouring the CP1 or CP2 mutations and assessed their impact on viral replication. The replication-promoting effect of these mutated HBx proteins was relatively low compared to the effects observed with the corresponding core promoter mutations. This finding is consistent with previous reports [30] and supports the interpretation that the core promoter sequence changes, rather than alterations in the HBx protein itself, play a primary role in regulating HBV transcriptional activity and replication efficiency.

A previous clinical study reported that the viral loads in HBeAg-negative chronic hepatitis B patients were 6.7±0.7, 2.6 and 4.5±2.2 log copies ml^−1^ for the 1757G/1762T/1764A, 1757A/1762T/1764A and 1764T/1766G mutation patterns, respectively [17]. Our current findings are largely consistent with those results, except HBeAg status: the 1757G/1762T/1764A and 1764T/1766G patterns demonstrated high replication efficiency, whereas the 1757A/1762T/1764A pattern exhibited markedly reduced replication compared to the 1757G/1762T/1764A pattern. These results support the notion that core promoter mutations can significantly influence viral replication levels, which may, in turn, contribute to differences in clinical outcomes among patients with chronic hepatitis B.

From a genetic perspective, the frequent co-occurrence of the 1757A substitution – a characteristic feature of HBV/D – with the CP2 mutation suggests that the 1757A mutation may have preceded the emergence of the CP1 and CP2 mutations. In contrast, in other HBV genotypes where the 1757A substitution is not commonly observed, the CP1 mutation appears to arise more frequently, implying that CP1 may be preferentially selected in the absence of 1757A. Moreover, it is plausible that the 1757A mutation evolved in response to selective pressures imposed by host genomic factors, such as immune responses. This possibility is particularly relevant in regions where HBV/D1 is endemic, including parts of India. Coevolution with human genetic or immunological backgrounds may have contributed to the establishment of this mutation. Nevertheless, further studies, including population-based and phylogenetic analyses, are required to clarify the evolutionary trajectory and functional significance of the 1757A substitution.

This study has several limitations, including the inability to comprehensively identify the transcription factor networks involved in the genomic region spanning from the 1757A site to the CP2 region. While it is well established that multiple transcription factors interact with the HBV core promoter [35], our analysis focused solely on HNF3, which is specifically associated with the CP2 mutation. Given that the 1757A substitution is located distally from the CP2 site, it is unlikely to directly influence HNF3 binding. Nonetheless, the observed co-occurrence of the 1757A and CP2 mutations, along with their synergistic enhancement of viral replication, suggests the possibility of differential transcription factor complex formation involving HNF1 and HNF3. These findings imply that the composition and cooperative interactions of transcription factors at the core promoter may be more diverse and context-dependent than previously appreciated. Future studies will aim to identify additional transcription factors involved in this regulatory network and to elucidate their roles in modulating HBV replication.

## Supplementary material

10.1099/jgv.0.002225 Uncited Supplementary Material 1.

## Abbreviations

BCP: base core promoter
CMV: cytomegalovirus
CP1: A1762T/G1764A
CP2: G1764T/C1766G
GAPDH: glyceraldehyde-3-phosphate dehydrogenase
HBcAg: hepatitis B core antigen
HBeAg: hepatitis B e-antigen
HBsAg: hepatitis B surface antigen
HBV: hepatitis B virus
HBV/D: HBV genotype D
HBx: hepatitis B X protein
HCC: hepatocellular carcinomas
HNF: hepatocyte nuclear factor
pcRNA: precore mRNA
pgRNA: pre-genomic RNA
SEAP: secreted alkaline phosphatase
