# Supplementary material for: Impact of a naturally occurring hepatitis B virus genotype D-specific core-promoter mutation on viral replication
Source: J Gen Virol. 2026 Feb 19;107(2):002225. doi: 10.1099/jgv.0.002225 (PMC12919851; doi:10.1099/jgv.0.002225)
Supplement: Uncited Supplementary Material 1. [file jgv-107-02225-s001.pdf]

Supplemental Information

Impact of a naturally occurring hepatitis B virus genotype D-specific core-promoter mutation on viral replication

Running title: Role of a hepatitis B virus genotype D-specific mutation

Masatake Kanai[1, 2], Tadasu Shin-I[1], Tomoko Date[1], Aiko Sakai[1], Masashi Mizokami[3], and Masaya Sugiyama[1, *]

1. Department of Viral Pathogenesis and Controls, National Institute of Global Health and Medicine, Japana Institute for Health and Security. Tokyo, Japan.

2. Laboratory of Macromolecular Biochemistry, Department of Chemistry for Life Sciences and Agriculture, Faculty of Life Science, Tokyo University of Agriculture. Tokyo, Japan.

3. Cellular & Molecular Biotechnology Research Institute, National Institute of Advanced Industrial Science and Technology. Ibaraki, Japan.

*To whom correspondence should be addressed:

Masaya Sugiyama Ph.D.,

Department of Viral Pathogenesis and Controls, National Institute of Global Health and Medicine, Japana Institute for Health and Security.

1-21-1, Toyama, Shinjuku, Tokyo 162-8655, Japan.

Tel:+81-(0)3-3202-7181

email: sugiyama.m@jihs.go.jp

1. **Supplemental Table**
2. **Supplemental Figure legend**
3. **Supplemental Figures**

Supplemental Table 1. Primer list used in this study

Name	Forward/ reverse	Sequence
1757A	F	GAGGAGATTAGATTAAAGGTCTTTG
1757A	R	CAAAGACCTTTAATCTAATCTCCTC
1762T	F	GAGGAGATTAGGTTAATGGTCTTTG
1762T	R	CAAAGACCATTAACCTAATCTCCTC
1764A	F	GAGGAGATTAGGTTAAAGATCTTTG
1764A	R	CAAAGATCTTTAACCTAATCTCCTC
1762T/64A	F	GAGGAGATTAGGTTAATGATCTTTG
1762T/64A	R	CAAAGATCATTAACCTAATCTCCTC
1757A/62T	F	GAGGAGATTAGATTAATGGTCTTTG
1757A/62T	R	CAAAGACCATTAATCTAATCTCCTC
1757A/64A	F	GAGGAGATTAGATTAAAGATCTTTG
1757A/64A	R	CAAAGATCTTTAATCTAATCTCCTC
1757A/62T/64A	F	GAGGAGATTAGATTAATGATCTTTG
1757A/62T/64A	R	CAAAGATCATTAATCTAATCTCCTC
1764T	F	GAGGAGATTAGGTTAAAGTTCTTTG
1764T	R	CAAAGAACTTTAACCTAATCTCCTC

1766G	F	GAGGAGATTAGGTTAAAGGTGTTTG
1766G	R	CAAACACCTTTAACCTAATCTCCTC
1764T/66G	F	GAGGAGATTAGGTTAAAGTTGTTTG
1764T/66G	R	CAAACAACTTTAACCTAATCTCCTC
1757A/64T	F	GAGGAGATTAGATTAAAGTTCTTTG
1757A/64T	R	CAAAGAACTTTAATCTAATCTCCTC
1757A/66G	F	GAGGAGATTAGATTAAAGGTGTTTG
1757A/66G	R	CAAACACCTTTAATCTAATCTCCTC
1757A/64T/66G	F	GAGGAGATTAGATTAAAGTTGTTTG
1757A/64T/66G	R	CAAACAACTTTAATCTAATCTCCTC
X- (*)	F	GCT AGG CTG TGC TGC TAA CTG GAT C
X- (*)	R	G ATC CAG TTA GCA GCA CAG CCT AGC
POL- (**)	F	G AGA CTA CTG TTG TAA GAC GAC GAG
POL- (**)	R	CTC GTC GTC TTA CAA CAG TAG TCT C

The underlined nucleotide indicates the site of the introduced mutation.

(*) This mutation introduces a stop codon in the HBx gene but does not affect the amino acid sequence of the overlapping polymerase (POL) gene, which is encoded in a different reading frame.

(**) This mutation introduces a stop codon in the POL gene but does not affect the amino acid sequence of the overlapping HBx gene, which is encoded in a different reading frame.

Supplemental Figure 1. Assessment of HBX sensitivity and dose-dependent effects of HBX expression on HBV replication in HepG2 cells

As a preliminary experiment for Figure 7, Southern blot analysis was performed to evaluate HBV replication following co-transfection of HBX and HBV/D expression plasmids into HepG2 cells. Because an antibody suitable for direct detection of HBX was not available in our laboratory, an HBX expression plasmid carrying a Myc tag was used.

(A) The sensitivity of HepG2 cells to HBX was first assessed. Wild-type pUC19/HBV/D and an HBX-deficient pUC19/HBV/D construct were prepared. In the HBX-deficient construct, expression of HBX restored viral replication efficiency to a level comparable to that of the wild-type pUC19/HBV/D, indicating that this experimental system is responsive to HBX.

(B) Reporter activity was evaluated in HepG2 cells. Expression vectors encoding wild-type HBx or HBx carrying the CP1 or CP2 mutations were prepared. For each HBx construct, a corresponding reporter plasmid containing the same core promoter sequence was used; these reporter plasmids were identical to those used in the experiments shown in Figure 4. Specifically, the wild-type HBx expression vector was paired with a reporter plasmid containing the wild-type core promoter, the CP1 HBx mutant with a reporter containing the CP1 core promoter, and the CP2 HBx mutant with a reporter containing

the CP2 core promoter. Using these three matched sets, the amount of HBx expression plasmid was varied. For comparison of luciferase activities, the activity obtained with 0 ng of the wild-type HBx expression plasmid was used as the reference value. Reporter activity increased in a dose-dependent manner with increasing amounts of HBx expression plasmid and was not significantly affected by the presence or absence of HBx mutations.

(C) The effect of HBX expression levels on HBV replication was then examined. Stepwise increases in the amount of the HBX expression plasmid resulted in a modest but dose-dependent increase in HBV replication.

A

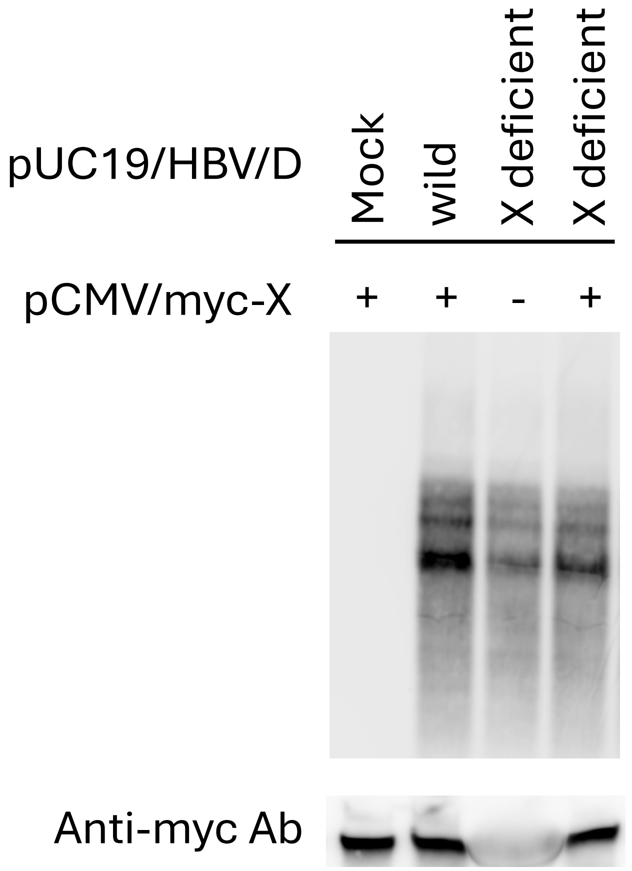

B

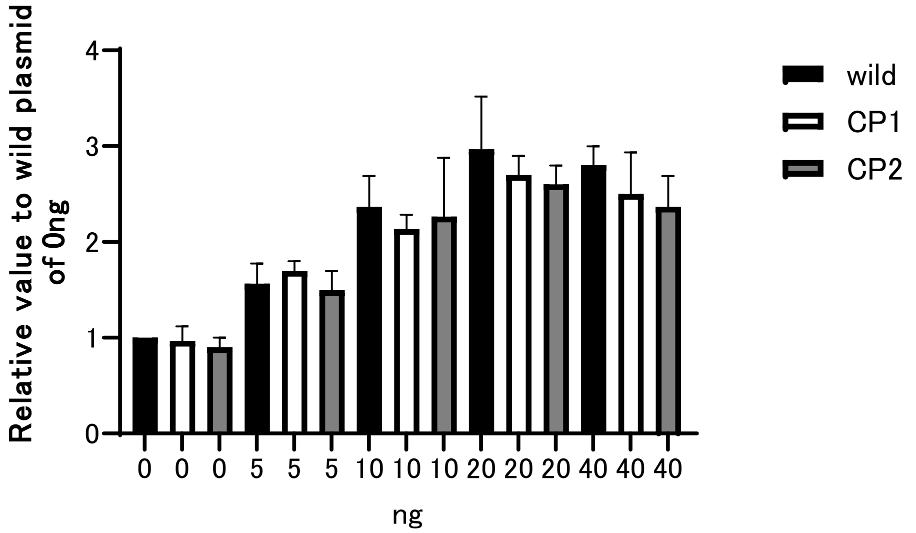

Supplemental Figure 1

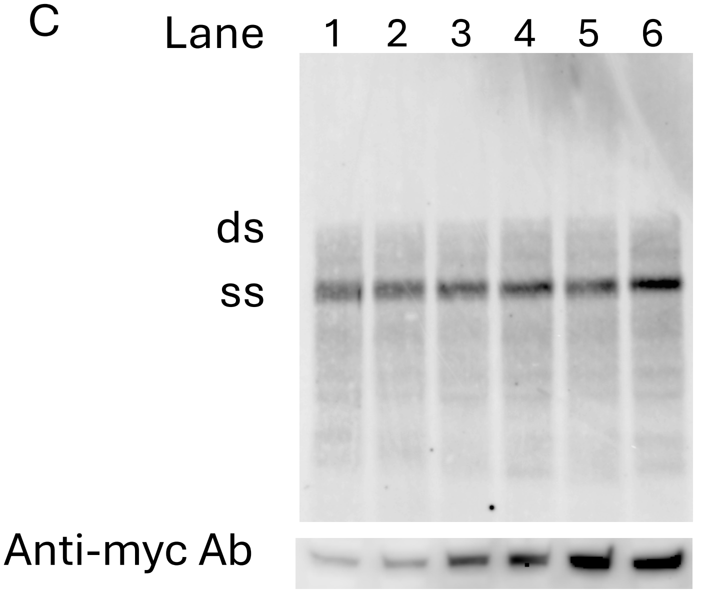

plasmid/ Lane	1	2	3	4	5	6	
pCMV/myc-X	10	50	100	250	500	1000	
pCMV/empty	990	950	900	750	500	0	(ng)

Supplemental Figure 1
